# Transforming Diabetes Care: The Molecular Pathways through Which GLP1-RAs Impact the Kidneys in Diabetic Kidney Disease

**DOI:** 10.3390/biomedicines12030657

**Published:** 2024-03-14

**Authors:** Merita Rroji, Goce Spasovski

**Affiliations:** 1 Department of Nephrology, Faculty of Medicine, University of Medicine Tirana, 1001 Tirana, Albania; 2 University Clinic for Nephrology, Medical Faculty, University St. Cyril and Methodius, 1000 Skopje, North Macedonia; spasovski.goce@gmail.com

**Keywords:** diabetic kidney disease, inflammation, natriuresis, renal protection

## Abstract

Diabetic kidney disease (DKD) is a substantial complication of type 2 diabetes (T2D), presenting challenges in chronic kidney disease (CKD) management. In addition to traditional and recent therapies, including angiotensin, converting enzyme (ACE) inhibitors, angiotensin receptor blockers, sodium-glucose cotransporter 2 (SGLT2) inhibitors, and mineralocorticoid receptor antagonists, the evolution of antihyperglycemic treatments has introduced a promising agent, glucagon-like peptide-1 receptor agonist (GLP-1RA) for the management of DKD. GLP-1RAs enhance insulin release and reduce glucagon release, offering a novel approach to DKD management. This review analyzes the molecular pathways through which GLP1-RAs confer renal protection in T2D and DKD, which are complex and multifaceted. They include modulation of renal hemodynamics, antioxidative and anti-inflammatory actions, metabolic regulation, and direct cellular effects. These mechanisms highlight GLP1-RA’s potential as a therapeutic option for glycemic control and direct or indirect renal function protection in diabetic patients, emphasizing the potentiality of GLP-1RAs for dual therapy, with cardiovascular and renal protection as a holistic approach. Clinical evidence supports GLP-1RAs in reducing albuminuria and enhancing kidney outcomes, highlighting their value in a comprehensive DKD management strategy.

## 1. Introduction

Exploring glucagon-like peptide-1 receptor agonist (GLP1-RA) in renal protection, especially in diabetic kidney disease (DKD), reveals a refined molecular landscape. GLP-1 therapies show a wide range of beneficial effects on kidney health. These effects have been observed in both diabetic and non-diabetic models of chronic kidney disease (CKD) and acute kidney injury (AKI).

Both animal and clinical studies have demonstrated the effectiveness of GLP-1-based treatments in improving kidney function. We discuss several mechanisms that have been proposed to explain the renal effects of GLP-1RAs, including direct actions on renal hemodynamics, reduction of systemic and intraglomerular pressure, modulation of tubuloglomerular feedback, inhibition of the oxidative stress, inflammation, and fibrosis.

### 1.1. Multifaceted Roles of GLP-1 in Metabolic Regulation

Glucagon-like peptide-1 (GLP-1), a critical hormone pivotal in glucose regulation, plays diverse roles in its metabolism. It originates from the preproglucagon (PPG) precursor encoded by the glucagon (GCG) gene. This gene, exhibiting expression in various body regions such as the pancreas, intestines, and central nervous system (CNS), orchestrates the synthesis of GLP-1 and other related peptide hormones. The generation of GLP-1 predominantly occurs via the post-translational modification of proglucagon, facilitated by proprotein convertase subtilisin/kexin type 1 or 3 in L cells of the terminal ileum and colon, as well as neurons in the nucleus of the solitary tract [1].

Nutrients like glucose, amino acids, free fatty acids, and bile acids stimulate GLP-1 secretion. These stimulants influence the hormone’s release through intracellular calcium and cAMP pathways. The regulatory mechanisms of GLP-1 secretion are complex and must be fully elucidated. Despite its pancreatic production, GLP-1’s contribution to circulating levels and receptor-mediated actions external to the pancreas is minimal. The hormone exerts its effects through the GLP-1 receptor, a member of the class B G protein-coupled receptor family, which is widespread across various tissues, underscoring GLP-1’s extensive role in systemic homeostasis, including glucose metabolism [2].

GLP-1’s primary metabolic functions are centered around the glucose-responsive enhancement of insulin secretion and the suppression of glucagon release from the pancreas. Here, it augments the insulin secretion, encourages beta-cell proliferation, hinders apoptosis, and modulates the release of other hormones, such as somatostatin and glucagon [3,4,5].

Further, GLP-1 influences gastric emptying and intestinal motility, affecting postprandial blood glucose levels, satiety, and food intake potentially contributing to weight loss. Existing in two bioactive forms, GLP-1 undergoes rapid cleavage by dipeptidyl peptidase 4 (DPP4), an enzyme targeting not only incretins but also various molecules with implications of immune cell function. GLP-1 degradation products, however, do not regulate glucose homeostasis through GLP-1 receptors as they lack ligand properties for these receptors. Neutral endopeptidase also processes GLP-1, yielding variants that potentially affect glucose utilization in the liver. The active GLP-1 fraction reaching target organs is minimal before the enzymatic cleavage and metabolism, primarily in the kidneys [6,7].

The swift degradation of GLP-1 by DPP4, leading to a brief half-life, has driven the development of GLP-1 receptor agonists (GLP-1RAs) as an efficacious approach in diabetes management.

### 1.2. GLP-1RA Development

GLP-1RAs effectively mimic the actions of natural GLP-1, significantly impacting glucose metabolism and weight management. They amplify insulin secretion, attenuate glucagon release, support beta-cell functionality, and enhance insulin sensitivity. GLP-1RAs also slow the gastric emptying, that combined with their metabolic effects, contributes to decreased hemoglobin A1c (HbA1c) levels and reduced fasting glucose concentrations, which in turn facilitates weight loss through prolonged gastric emptying and increased satiety [8,9].

A pioneering approach in this realm involved using exendin-4, a compound sourced from the Gila monster’s saliva (Heloderma suspectum). Exendin-4 shares a structural similarity with GLP-1, sufficient to activate the GLP-1 receptor, yet exhibits resistance to degradation by DPP4. This discovery paved the way for pharmaceuticals like exenatide and lixisenatide. However, these exendin-4-based therapies are subject to renal elimination and necessitate regular administration due to their limited half-life [10].

Addressing this challenge, researchers have modified the human GLP-1 molecule to increase its resistance to DPP4 degradation and reduce renal clearance [1]. Innovative formulations have been developed, such as the covalent or noncovalent albumin binding found in albiglutide, semaglutide, and liraglutide or the attachment to antibody fragment crystallizable domains evident in dulaglutide.

The therapeutic impact of GLP-1RAs varies depending on their action duration. Short-acting agents like exenatide and lixisenatide intermittently activate GLP1 receptors, closely mirroring the physiological patterns of natural GLP1. This preserves their capacity to modulate gastric emptying and influence postprandial glucose levels. In contrast, long-acting GLP-1RAs, such as liraglutide, exenatide XR, albiglutide, dulaglutide, and liraglutide, ensure continuous receptor engagement. While superior in the management of fasting glucose and HbA1c levels, their persistent receptor stimulation may lead to tachyphylaxis, potentially diminishing their effectiveness in delaying gastric emptying. Nevertheless, both short- and long-acting GLP-1RAs demonstrate comparable efficacy in weight reduction [11].

Moreover, exenatide has been innovatively formulated into an extended-release microsphere, enabling slower release and less frequent dosing. This advancement has led to most GLP-1RAs requiring only weekly injections, except liraglutide, which is administered daily [12].

GLP-1RAs are now advised as the initial choice for injectable glucose-lowering treatments in type 2 diabetes due to their comparable or even better capacity to decrease HbA1c levels, coupled with the benefits of weight loss and a lack of inherent risk for hypoglycemic events, potentially even preceding the use of insulin. Additionally, it is possible to use GLP-1RAs in conjunction with basal insulin, available in separate and combined dosages. Notably, newer drugs such as semaglutide have demonstrated a superior effectiveness in reducing blood glucose levels aiding in weight loss [1,8].

A notable breakthrough in GLP-1RA therapy was the introduction of an orally administered version of the drug, specifically, oral semaglutide. Achieved by combining the medication with sodium N-(8-[2-hydroxybenzoyl]amino)caprylate(SNAC), this innovation elevates stomach pH levels and impedes pepsin activity, safeguarding the drug from degradation and enhancing its absorption [13].

### 1.3. Localization and Expression of Glucagon-like Peptide-1 Receptors in Renal Tissues

Identifying the glucagon-like peptide-1 receptor (GLP-1R) in 1992 has been pivotal in advancing the comprehension of its anatomical distribution, particularly within the renal system [14]. Various methodologies, such as RT-PCR and in situ hybridization, have been employed to detect GLP-1R mRNA in this way to indicate gene transcription. However, these techniques have limitations in confirming the presence of functional GLP-1Rs, as the correlation between mRNA and protein levels might be inconsistent [15].

Studies across humans, mouse, and rat models have confirmed the presence of GLP-1R transcripts in kidney tissues. However, the specificity of these findings at the cellular level has been constrained due to the utilization of homogenized kidney samples. Focused research has identified GLP-1R mRNA in rat glomeruli and proximal convoluted tubules, as well as in the mouse glomerular capillaries and renal arteries [16].

An alternative method, employing radioactively labeled GLP-1, detected GLP-1R in human renal arteries but not within the tubular system. Initial studies using commercially available polyclonal antibodies indicated the presence of GLP-1R in various kidney regions, including the glomeruli and renal arteries. However, these antibodies were later identified as having low specificity [17].

A monoclonal antibody specifically targeting the extracellular domain of the GLP-1R was developed to enhance precision in detecting these receptors. This antibody showed increased specificity, selectively staining cells transfected with GLP-1R, facilitating a more accurate localization of these receptors. Immunohistochemistry studies leveraging this antibody have revealed that GLP-1R is predominantly located in smooth muscle cells within the renal vasculature rather than in tubular segments or endothelial cells, underscoring its specific distribution in these areas [18]. Further research utilizing this extensively validated monoclonal antibody has provided novel insights into GLP-1R localization, indicating its exclusive expression in both monkeys’ and humans’ preglomerular vascular smooth muscle cells and juxtaglomerular cells [19]. However, the precise intracellular localization of GLP-1R remains to be fully elucidated [18,19]. Additionally, GLP-1R has been identified as not being uniformly distributed within the vascular system [20,21]. Techniques such as in vivo autoradiography, immunohistochemistry, and in situ hybridization have identified GLP-1R in the arterial VSMCs of the kidneys in rats, mice, and monkeys, but only within a limited group of cells [18].

There is a significant gap in our understanding of how these receptors behave in live organisms compared to cell cultures [22,23]. Jensen et al. [24] discovered that GLP-1R is located in the renal microcirculation, i.e., in afferent arterioles. The experiments on rodents showed specific receptor binding, and in rats, GLP-1 infusion has led to an increased blood pressure, renal blood flow, and urine flow. Furthermore, it was found that GLP-1 and exendin-4, a GLP-1 analog, impacted afferent arterioles’ responses to pressure changes in isolated mouse kidneys, indicating a role in modulating renal blood flow [6].

GLP-1R activation in vascular smooth muscle cells increases cyclic AMP (cAMP), which in turn activates the activation of protein kinase A (PKA) and Exchange protein directly Activated by cAMP (EPAC). These pathways cause vasodilation by activating various potassium channels at the same time increasing the calcium release from the sarcoplasmic reticulum. This process is similar to GLP-1R’s function in pancreatic cells. The mechanisms by which GLP-1R activation influences renal vasodilation still need to be fully understood despite its observed effects on different arterial systems [15].

The inconsistent detection of GLP-1R in renal blood vessels, particularly in VSMCs, complicates our understanding of GLP-1’s role in kidney function, i.e., the body’s handling of albumin, water, and salt, either directly or through the kidneys and other mechanisms. Thus, the exact role of GLP-1 in both vascular and kidney health remains a topic that needs further study and clarification.

## 2. The Role of GLP-1 Receptor Agonists in Renal Protection and Diabetic Kidney Disease Management

GLP-1RAs have attracted considerable interest for their potential effects on renal hemodynamics and their role in reducing glomerular hyperfiltration. These pharmaceutical agents successfully inhibited hyperfiltration and ameliorated albuminuria while concurrently attenuating renal inflammation and oxidative stress. Furthermore, they played a pivotal role in preserving the glomerular filtration rate (GFR) exhibiting significant reduction in the histopathological manifestations associated with diabetic nephropathy [5].

### 2.1. Understanding the Complex Pathophysiology of Diabetic Kidney Disease: Identifying Molecular Sites for Therapeutic Interventions

DKD is a common and severe complication in individuals with diabetes, affecting about one-third of those diagnosed. The presence of pathological levels of albuminuria, diabetic glomerular lesions, and a decline in GFR characterizes it. The pathophysiology of DKD progression is complex and involves multiple molecular pathways. These pathways, including inflammatory processes, oxidative stress, structural changes within the kidneys, alterations in blood flow dynamics, and metabolic disorders, trigger harmful physiological events [25].

High glucose levels stimulate the production of growth factors, such as transforming growth factor-β (TGF-β) and connective tissue growth factor (CTGF), which are the key mediators in mesangial cell proliferation and matrix expansion. These growth factors activate mesangial cells to produce excessive extracellular matrix components, leading to glomerulosclerosis, a hallmark of DKD characterized by thickening of the glomerular basement membrane and expansion of the mesangial matrix. This proliferation disrupts the typical architecture and function of the glomerulus and impairs filtration, contributing to the progressive decline in renal function seen in DKD [26].

Hyperglycemia plays a significant role in the pathogenesis of DKD, triggering a complex network of mechanisms leading to renal damage. The process begins with the formation of advanced glycation end products (AGEs) and the activation of their receptor (RAGE), initiating a cascade of signaling events, including the upregulation of protein kinase C, nuclear factor-kappa B, and transforming growth factor-β (TGF-β). This chain of events leads to the increased production of reactive oxygen species (ROS) and a chronic inflammatory response, pivotal in the development and progression of DKD [27].

RAGE activation further contributes to renal damage by promoting glomerular matrix production and oxidative stress through mitochondrial superoxide production. It also induces the epithelial–mesenchymal transition of renal tubular cells, leading to interstitial fibrosis. Experimental studies highlight RAGE-mediated mitochondrial dysfunction, often via NAD(P)H oxidase activation, as an early indicator of DKD, preceding clinical manifestations such as albuminuria and histological changes. Despite these insights, clinical trials focusing on oxidative stress and inflammation have not yet confirmed the effectiveness of targeted therapies, underscoring the importance of strict glucose control [28].

In the early stages of diabetes, there is a notable increase in intraglomerular pressure coupled with hyperfiltration, which is pivotal in the genesis and exacerbation of DKD. This hyperfiltration phenomenon can be partly attributed to anomalies in tubuloglomerular feedback mechanisms [28,29]. Abnormalities in the feedback mechanisms between different kidney parts can partially explain this increased filtration. During hyperglycemia, glucose filtration increases, leading to more glucose and sodium reabsorbing in the early part of the kidney’s tubules. This alteration reduces the amount of sodium reaching the distal tubular macula densa, decreasing afferent arteriolar resistance and raising intraglomerular pressure. Furthermore, the imbalance in vasoactive substances such as angiotensin II and endothelin-1 play a significant role in this dysregulation [30].

Glomerular hypertension exerts substantial mechanical stress on the capillary walls, being a precursor to glomerulosclerosis and the depletion of peritubular capillaries. Moreover, it facilitates increased protein filtration into the tubular lumen, which triggers the synthesis of proinflammatory and profibrotic factors with accelerated kidney damage [31].

In addition, diabetes and hyperglycemia increase the energy that cells in the kidney tubules need to enhance glucose reabsorption and increase filtration. At the same time, the oxygen supply to the kidneys is reduced in DKD due to the loss of peritubular capillaries and development of fibrosis (scarring) in the kidney’s tissue. This mismatch between oxygen demand and supply leads to hypoxia that worsens kidney damage by promoting inflammation, oxidative stress, impairment of cellular waste removal processes (autophagy), and further fibrosis. DKD animal models have demonstrated that interventions to decrease hypoxia can improve renal function [32].

Recent studies have also highlighted the importance of autophagy, a critical cellular process for maintaining balance within cells by breaking down large molecules and organelles. Impaired autophagy, especially in podocytes, exacerbates kidney damage under diabetic conditions. Additionally, epigenetics, which refers to changes in gene expression that occur without alterations in the DNA sequence, has gained attention in understanding DKD. This genetic memory leads to a continued expression of genes and phenotypes associated with high blood sugar levels, even after blood sugar normalization, indicating the complex and enduring impact of diabetes on kidney health and DKD progression [33,34].

Recently, researchers have been investigating how medications known as GLP-1RAs, primarily used in treating type 2 diabetes, might influence the kidneys and affect DKD progression [35]. The use of GLP-1RAs has been associated with improved kidney outcomes, including reduced albuminuria and preservation of the filtration rate. These findings have sparked interest in the potential kidney-protective benefits of GLP-1RAs for patients with DKD [36,37].

### 2.2. Impact of Glycemic Control and GLP-1 Receptor Agonists on CKD Progression: Mitigating Hyperfiltration and Albuminuria in Diabetes

Hyperfiltration, identified as an initial marker of DKD, is intricately linked to hyperglycemia [38]. The pathophysiological bridge between hyperglycemia and hyperfiltration predominantly involves the upregulation of sodium reabsorption via the SGLT-2 cotransporters, leading to adjustments in tubuloglomerular feedback and subsequently modulating the blood flow within the glomerular afferent arterioles. The efficacy of strict glucose regulation in mitigating the incidence of microalbuminuria and macroalbuminuria underscores its significance as a cornerstone in managing early CKD stages.

Intensive glycemic management reverses hyperfiltration, particularly in type 1 diabetes mellitus (T1DM) with insulin therapy and well controlled blood glucose levels. In addition to the improvement in hyperfiltration state, the therapy significantly reduces the risk and progression of albuminuria as a critical marker of nephropathy. Namely, the risk of microalbuminuria and albuminuria appearance has been reduced by 39 and 54 percent, respectively [39].

Furthermore, the intensive glycemic control has delayed the onset and progression of an early stage of diabetic microvascular complications in Japanese patients with T2D and the progression of nephropathy [40]. The discussion extends to the potential benefits of GLP-1RAs in the prevention of DKD going beyond glycemic control. Indeed, their mechanistic pathways of renal protection include improved metabolic control and modulated inflammatory response, retarding the progression to macroalbuminuria and thus, preserving kidney function.

Clinical trials have shown the safety and efficacy of GLP-1RAs in patients with moderate to severe kidney dysfunction, improving blood sugar control and reducing body weight. Studies like HARMONY 8 [41], LIRA-RENAL [42], AWARD-7 [43], and PIONEER 5 [44] compared GLP-1RAs to other treatments such as sitagliptin, placebo, or insulin glargine, demonstrating GLP-1RAs’ superiority or comparable efficacy in controlling glycemia. Although these trials indicated positive outcomes on surrogate kidney markers like albuminuria with no harmful effects on kidney function, they were not designed to conclusively determine the impact on significant kidney outcomes due to their short duration and limited power. GLP-1RAs, such as liraglutide [42] and semaglutide [45], have been shown to significantly reduce the progression of macroalbuminuria among diabetic patients, indicating a potential protective effect on kidney function without markedly altering the eGFR. This observation suggests that while GLP-1RAs effectively slow the progress to more severe stages of albuminuria, their impact on overall kidney disease progression in diabetic patients might be greater than changes in albuminuria levels. Indeed, further analysis and data suggest that GLP-1RAs offer broader renal protection by potentially reducing albuminuria (both macro- and microalbuminuria) and slowing the decline in kidney function over time [5,46].

### 2.3. Exploring the Influence of GLP-1 Receptor Agonists on Renal Hemodynamic

GLP-1RAs have attracted considerable scholarly interest for their prospective influence on renal hemodynamics. These agents attenuate glomerular hyperfiltration, a process facilitated by their capacity to increase diuresis and natriuresis.

#### 2.3.1. Exploring the Dynamic Role of GLP-1R and GLP-1RA in Renal Natriuresis and Renal Hemodynamic: Acute and Chronic Perspectives

Exploring the dynamic role of GLP-1R and GLP-1RA unveils their significant impact on renal natriuresis and hemodynamics. In addition, the tubular effects of GLP-1R activation, acute and chronic responses, and the mechanisms driving these responses have been investigated.

Experimental and human studies have elucidated the crucial role of GLP-1 in modulating renal physiology, mainly through its tubular effects that induce natriuresis and diuresis. This mechanism primarily involves the inhibition of the Na+/H+ exchanger 3 (NHE3) in the proximal tubules, leading to decreased sodium reabsorption and enhanced natriuresis. GLP-1 exerts this effect by binding to its receptor, which activates PKA and results in the phosphorylation of NHE3, thereby diminishing sodium reabsorption. The observation that GLP-1 receptors are absent in proximal tubules suggests that the natriuretic responses to GLP-1-targeted therapies are likely mediated through indirect mechanisms rather than direct effects on these tubules. Additionally, GLP-1RAs have been shown to potentially increase renal blood flow and decrease vascular resistance, effects attributed to the stimulation of local nitric oxide production [15,24,47,48].

In research studies with anesthetized rats and various animal models, acute activation of GLP-1R reduces sodium reabsorption in the proximal tubules with a less marked effect in spontaneously hypertensive rats, suggesting these effects might also be due to GLP-1R located outside the kidney or other natriuretic mechanisms [49]. Data from human studies also present inconsistent results, indicating possible variations in the experimental setups [50,51]. Thus, the relationship between GLP-1RAs-induced natriuresis and its kidney-protective effects might be considered through the activation of tubuloglomerular feedback. This mechanism potentially reduces glomerular hyperfiltration and pressure stemming from increased distal sodium chloride delivery due to decreased NHE3 activity. Although this effect has been shown in standard rat models, clinical studies in patients with T2D, with or without DKD, have inconsistently shown an acute reduction in eGFR following GLP-1RA administration [51,52].

Nonetheless, a notable and consistent finding from these studies is a significant decrease in albuminuria, suggesting a connection between enhanced sodium transport to the distal nephron, increased afferent arteriolar resistance, reduced intraglomerular pressure, and the prevention of hyperfiltration, which is a common early feature of diabetic nephropathy [53,54].

Furthermore, GLP-1Rs have been identified within the renin-secreting cells of the juxtaglomerular apparatus, a critical area for blood pressure regulation. It is reported that when GLP-1 is infused into humans, it enhances natriuresis—the process by which the kidneys excrete sodium into the urine—without significantly impacting renin secretion [55]. This observation suggests that GLP-1 may contribute to lowering blood pressure through mechanisms that bypass the direct activation of the renin system [56].

Additionally, the role of extrarenal GLP-1Rs and a general increase in cAMP production following GLP-1R activation might contribute to natriuresis, paralleling the kidney’s response to glucagon. GLP-1R-expressing neurons in the brain are also considered to potentially influence water and salt balance [57,58].

Interestingly, GLP-1 infusion activates GLP-1R present in the walls of the renal pelvis and stimulates renal afferent nerves increasing renal sympathetic nerve activity (RSNA), mean arterial pressure (MAP), and heart rate (HR), mediating the diuretic and natriuretic effects of GLP-1 by the neural pathways. Furthermore, approaches like therapeutic renal denervation (T-RDN) and selective afferent renal denervation (A-RDN) were shown to enhance these effects while moderating the associated pressure and tachycardic responses [59].

These observations underscore a complex interaction of GLP-1 with renal nerves in regulating the sodium and water balance, essential for maintaining fluid balance and blood pressure stability. Additionally, the tubular effects of GLP-1R activation involve a blend of direct and indirect mechanisms, implicating the nervous system, renin–angiotensin system (RAS), and the regulation of atrial natriuretic peptide (ANP) [60,61].

Regarding chronic effects, GLP-1RA like lixisenatide was shown to reduce NHE3 activity in overweight T2D patients, leading to an increased natriuresis over eight weeks. Nevertheless, long-term GLP-1RA treatment has only slightly increased sodium excretion in people with type 2 diabetes, raising questions about the long-term impact of GLP-1R activation on the kidneys’ sodium handling and blood pressure control [15]. This has led to speculation about possible GLP-1R desensitization over time.

Although a direct link between GLP-1RA-induced natriuresis and kidney-protective effects is not well established, the presence of GLP-1R in the vascular smooth muscle cells of the afferent arteriole may be viewed as a key site for autoregulatory resistance changes. Thus, any direct renal vasodilatory effect of GLP-1 could impair renal autoregulation, negatively impacting renal function.

#### 2.3.2. Effects on Renal Autoregulation

Renal autoregulation involves at least two main mechanisms: the myogenic response and tubuloglomerular feedback (TGF). Both mechanisms operate on the afferent arteriole and the distal part of radial arteries, contributing to the primary pressure drop in the kidneys. The myogenic response, intrinsic to vascular smooth muscle cells, causes constriction in response to increased intravascular pressure. The exact mechanism linking increased wall stress to constriction has yet to be fully understood. Presently, it is known that depolarization as a part of the process may be inhibited by antagonists of voltage-operated Ca^2+^ channels. Additionally, the transient receptor potential and epithelial Na^+^ channels are implicated in this depolarization [62,63,64].

TGF is a critical regulatory mechanism in the kidneys, initiated when the macula densa cells detect an increase in sodium chloride (NaCl) concentration in the filtrate at the junction of the loop of Henle and the distal convoluted tubule, indicating elevated GFR. In response, these cells release signaling molecules like adenosine and ATP, which cause the nearby afferent arteriole to constrict, thereby reducing blood flow into the glomerulus and decreasing GFR towards normal levels. Although primarily a local mechanism, the vasoconstriction effects of TGF can influence adjacent vascular structures and potentially synchronize responses across multiple nephrons through gap junctions. This interaction, along with the myogenic response—a separate mechanism that adjusts vessel tone in response to blood pressure changes—ensures stable renal perfusion and filtration rates, illustrating the intricate balance of renal regulatory processes to maintain systemic homeostasis [15].

The effect of GLP-1RA on renal function varies across different health conditions. Increased lithium clearance following GLP-1R activation in humans [56] and rodents [65,66] suggests enhanced outflow from the proximal tubule, indicating that TGF activation may counterbalance the increased tubular flow, potentially leading to a decrease in GFR. While acute GLP-1 treatment in animal studies appears to attenuate the myogenic response, leading to afferent arterioles’ dilatation, in type 2 diabetic patients with normal kidney function, the short-term treatment with GLP-1Ra did not significantly affect GFR, suggesting preservation of the myogenic response and renal autoregulation [67].

The effects of an extended GLP-1R activation on renal autoregulation in type 2 diabetes remain to be determined [68]. At an early stage of DKD, it is postulated that more significant sodium reabsorption through SGLT2 and unresponsiveness of TGF leads to hyperfiltration. Thus, a possible early effect of GLP-1 may attenuate this hyperfiltration by reducing sodium reabsorption in the proximal tubule. Hence, the stabilization or enhancement of GFR by GLP-1 treatment in the initial phases of renal impairment may be plausible. However, with the progression of kidney disease and increasing loss of nephrons that impaired GFR, the impact on reduction in proximal reabsorption by GLP-1R agonists may be questionable.

In placebo-controlled trials of type 2 diabetes patients with moderate to severe CKD, GLP-1R agonists significantly slowed the decline in eGFR. This could indicate that the vasodilatory aspects attained by the activation of GLP-1R could outweigh the TGF activation [43].

### 2.4. GLP-1RAs: Effect on Blood Pressure Control in Type 2 Diabetes

GLP-1RAs may help in the blood pressure control through multiple mechanisms, impacting the central nervous and peripheral systems. The GLP-1 receptor, found in essential brain areas like the hypothalamus and brainstem, regulates sympathetic nervous system activity, often enhanced in hypertension.

Along with central mechanisms, GLP-1Rs in the vascular system, such as those found in endothelial cells and vascular smooth muscle cells, help maintain the tone of the vascular system. It is primarily achieved through signaling pathways that enhance vasodilation, helping to lower blood pressure [69,70]. Specifically, GLP-1 signaling in the vascular system supports endothelium-mediated vasodilation [71], indicating an integral role in cardiovascular health [72].

Another critical aspect of GLP-1RAs’ effect on blood pressure is related to their action on renal function. Through the promotion of natriuresis, these agents assist in the regulation of the extracellular fluid volume, directly influencing blood pressure control that is confirmed in experimental studies [52]. In addition, GLP-1RAs influence blood pressure control through short- and long-term effects, impacting renal function, vascular tone, and insulin sensitivity. Acutely, GLP-1RAs induce diuresis and natriuresis in parallel with the increasing GFR and inhibition of the sodium reabsorption in the kidneys, thereby supporting a role in the maintenance of the sodium balance and prevention of volume expansion. Conversely, GLP-1R blockade leads to decreased GFR and increased sodium reabsorption, suggesting that endogenous GLP-1R signaling helps regulate physiological blood pressure homeostasis. Long-term, GLP-1R blockade raises blood pressure and worsens renal outcomes in hypertensive subjects, confirming GLP-1R activity in the modulation of renal sodium handling, the renin–angiotensin system (RAS), and insulin sensitivity [73].

Overall, GLP-1RAs may offer a multifaceted approach to hypertension management targeting the nervous system in reducing sympathetic activity, enhancing vascular function for better blood flow, and improving renal sodium handling to stabilize fluid balance. Additionally, their potential to counteract systems like the renin–angiotensin system (RAS) and the sympathetic nervous system (SNS), additionally underscores their role in mitigating hypertension. These combined actions make GLP-1RAs a promising therapeutic option for blood pressure control, particularly in metabolic disorders [52,74].

Recent meta-analysis suggests that semaglutide, a GLP-1 receptor agonist, significantly lowers systolic blood pressure (SBP) in individuals with T2D, independent of its blood glucose-lowering effects. Despite these known benefits, it remains unclear whether semaglutide can also regulate the dysregulation of blood pressure’s circadian rhythm, and what its long-term effects are [75].

Nevertheless, beyond glycemic and blood pressure control, other potential mechanisms for preserving renal function with a GLP-1R agonist treatment include anti-inflammatory actions, reduced fibrosis, and reduced glomerular sclerosis [15].

### 2.5. GLP-1 Receptor Agonists: Bridging Antioxidative and Anti-Inflammatory Mechanisms in Diabetes, Atherosclerosis, and Kidney Function

Recent evidence underscores the pivotal role of GLP-1RA in mitigating oxidative stress and inflammation, which is crucial in managing diabetes, particularly for DKD and CV health. GLP-1RA plays a significant role in glucose metabolism and significantly reduces oxidative stress markers through the receptor-mediated activation of pathways such as cAMP, PI3K, and PKC. This leads to the enhanced activation of Nrf-2 and strengthens the body’s antioxidant defenses, which is crucial in diabetes, where there is an increase in free radicals and a weakened antioxidant defense [76,77]. Specifically, GLP-1Ra therapies have been shown to reverse hepatic steatosis and improve insulin sensitivity in diabetic models, highlighting their antioxidative efficacy. These findings are supported by in vitro and in vivo studies demonstrating GLP-1’s protective action against oxidative damage and its essential role in preventing diabetes-related complications, such as cardiac remodeling and cardiovascular disease. Importantly, GLP-1 plays a crucial role in oxidative stress reduction within tissues, restoring mitochondrial functions, and preventing oxidative harm. Such beneficial impacts contribute to averting the loss of podocytes and dysfunction in mesangial and endothelial cells [5,15,78].

Recent studies underscore the beneficial effects of GLP-1RAs on oxidative stress mechanisms in diabetic nephropathy, potentially curbing CKD progression. Specifically, liraglutide has been shown to elevate levels of critical antioxidative enzymes, such as catalase and glutathione peroxidase-3, in Streptozotocin (STZ)-induced diabetic mice, indicating its protective role against tissue oxidative stress [79]. Likewise, lixisenatide enhanced renal tissue’s total antioxidant capacity and reduced oxidative stress markers, including Malondialdehyde (MDA), alongside the normalization of iNOS and COX-2 expressions in a high-fat diet mouse model [80]. Exendin-4’s role extends to inhibiting increases in TGFβ, type 1 collagen, and fibronectin mRNA in diabetic mice’s kidneys, markers indicative of DKD [81]. Furthermore, liraglutide reduced superoxide production and normalized NADPH oxidase complex components in STZ-induced diabetic animals, correlating with reduced albuminuria—a finding supported by in vitro studies on human mesangial cells [82]. Additionally, the combined treatment of exenatide and olmesartan in obese, insulin-resistant rats showed a significant decrease in Nox4 expression and increased antioxidative enzymes, effectively reducing albuminuria [83]. GLP-1RAs also counteract fibrosis and reduce extracellular vesicle secretion in tubular epithelial cells under high-glucose conditions, protecting against ischemia-reperfusion injury by mitigating oxidative stress and inflammation [84,85].

The interaction between the immune system and DKD has become increasingly evident, with kidney biopsies revealing accumulations of immune cells, notably macrophages and T-cells, and elevated levels of proinflammatory cytokines (TNF-alpha, MCP-1/CCL2, IL-6, IL-1β), orchestrated by NF-κβ [86]. This inflammatory cascade, fueled by high glucose levels and AGEs, activates pattern recognition receptors, leading to chronic inflammation and mitochondrial dysfunction, culminating in fibrosis and end-stage kidney disease [86]. GLP-1RAs have emerged as a significant counterforce, mimicking the effects of endogenous GLP-1 by activating GLP-1 receptors, which leads to a reduction in proinflammatory responses and direct beneficial impacts on kidney and vascular tissues. In DKD, GLP-1RAs like liraglutide mitigate renal inflammation by decreasing macrophage infiltration and inflammatory markers [5,15] while also demonstrating efficacy in non-diabetic kidney injury models and diseases characterized by inflammation, such as asthma and psoriasis [87].

The role of GLP-1RAs extends to vascular inflammation, where they reduce endothelial dysfunction and atherosclerosis by dampening the presentation of adhesion molecules and reducing oxidative stress through the AMPK and MAPK pathways [87], along with enhancing Sirtuin-6 expression, thereby modulating inflammatory responses in cardiovascular diseases [88]. Notably, GLP-1-based therapies have been shown to ameliorate cardiac dysfunction in disease models by inhibiting proinflammatory mediators [53,89]. Beyond their anti-inflammatory effects, these therapies improve insulin sensitivity and lipid metabolism, addressing critical aspects of diabetes management and reducing cardiovascular risk [90,91,92,93,94,95]. Liraglutide, in particular, has been highlighted for its atheroprotective effects, stabilizing plaques, improving endothelial function, and directly inhibiting atherosclerotic lesion development [96,97,98,99,100]. Table 1 summarizes the antioxidative and anti-inflammatory mechanisms of diabetes and its impact on atherosclerosis and kidney function.

### 2.6. Targeting Obesity-Induced CKD: The Multifaceted Role of GLP-1R Agonists in Metabolic and Renal Health

Obesity is a significant contributor to CKD, highlighting the importance of treating individuals with obesity and CKD to reduce its associated morbidity. The causal relationship between obesity and CKD has been validated through Mendelian randomization analyses, which utilize genetic risk scores to demonstrate the impact of BMI on CKD and arterial stiffness [103]. These genetic studies, including analyses from the UK Biobank, point to both central and general adiposity as significant, independent factors in CKD causation, with diabetes, blood pressure, and their correlates being significant explanatory variables [42]. This body of evidence highlights the importance of weight management in the primary prevention and control of CKD and subclinical vascular diseases, underlining obesity’s significant role across various kidney disorders and its largely independent effects on blood pressure and type 2 diabetes [104].

The pathophysiology behind obesity-related kidney disease is multifaceted, involving adipose tissue accumulation that affects kidney hemodynamics, leading to conditions such as glomerulomegaly and fibrosis. Adiposity impacts inflammatory adipokines, affecting renal function through mechanisms like leptin-induced hypertension and adiponectin-related podocyte dysfunction [105]. Furthermore, obesity’s influence on the renin–angiotensin–aldosterone system and the lipotoxic activity within the kidney underscores the complex interaction between obesity and renal health. The exacerbation of CKD by type 2 diabetes (T2D), an obesity complication, further complicates the picture. T2D accelerates kidney damage through insulin resistance, affecting podocyte function and promoting oxidative stress and fibrosis. Insulin resistance in T2D leads to increased insulin secretion, growth factor release, and dysregulation of the glomerular filtration barrier, culminating in the obesity-related glomerulopathy and further kidney injury [106,107].

This intricate web of associations between obesity, T2D, and CKD highlights the urgent need for integrated approaches for the management of these conditions. In this context, the therapeutic potential of GLP-1R agonists emerges as a beacon of hope [108]. Previous research has established that such agonists can significantly reduce food intake, body weight, and blood glucose levels in obese rats. Consistent with these findings, it was shown that GLP-1RA effectively reverses the metabolic disorders induced by a high-fat diet (HFD), including the alleviation of elevated body weight, hyperlipidemia, and impaired glucose tolerance [106]. Hence, GLP-1 receptor agonists could help fight obesity and its complications partially by increasing the number of fat cells and converting white fat to beige fat, which burns more calories. This increases energy use, aiding in weight control and metabolic health. Another significant mechanism is GLP-1RA’s capacity to mitigate tubular damage and inflammation within the kidneys. Research using the db/db mouse model, which simulates obesity, has shown increased levels of GLP1 can reduce histological signs of tubulointerstitial damage and decrease the renal expression of proinflammatory markers such as TNFα and Ccl5. This reduction in inflammation is accompanied by a decrease in the infiltration of CD3+ T cells and F4/80+ macrophages into the kidney [102]. Mice with elevated GLP1 expression also demonstrate a notable improvement in survival rates.

Furthermore, studies have identified that the absence of GLP1R can lead to spontaneous kidney damage, including albuminuria and glomerulosclerosis, conditions that worsen in the context of type-1 diabetes. However, treatment with liraglutide, a type of GLP-1RA, has been shown to enhance podocyte structure and reduce albuminuria and glomerulosclerosis [109]. Furthermore, GLP-1RA’s renal protective role is highlighted through its capacity to improve renal function, ameliorate histological injury, and diminish the expression of proinflammatory and profibrotic factors in the kidneys of HFD rats. The development of GLP1 analogs, inspired by the understanding of the metabolic roles of GLP1 and exendin-4 (exenatide), has led to the creation of advanced formulations for steady administration and a prolonged effect. Among these products, liraglutide (Victoza, Saxenda), dulaglutide (Trulicity), and semaglutide (Ozempic) have received approval from the FDA. Initially prescribed for T2D patients, liraglutide and semaglutide have also gained authorization for obesity management [108,110]. Moreover, semaglutide has been recently approved for lowering cardiovascular event risks in T2D patients with established cardiovascular disease [108]. Additionally, these medications have shown effectiveness in lowering renal dysfunction and fatty liver disease.

## 3. Clinical Studies

In the treatment landscape for T2D with high cardiovascular risk, GLP1-RA have been the subject of numerous large-scale trials, underscoring their potential in kidney protection, including DKD. These trials have collectively contributed to a deeper understanding of the renal effects of GLP1-RA focused on the effects on albuminuria and eGFR.

The ELIXA Trial (Lixisenatide) was focused on T2D patients with recent acute coronary events. It was found that lixisenatide significantly reduced the urinary albumin-to-creatinine ratio (UACR) at 108 weeks. Despite this positive outcome, no significant change was observed in eGFR or other hard renal endpoints [111].

The LEADER and SUSTAIN-6 Trials with Liraglutide and Semaglutide, respectively, showed significant reductions in macroalbuminuria in individuals with a high cardiovascular risk profile. Importantly, these drugs also induced a slightly slower decline in eGFR compared to the placebo, an effect more pronounced in patients with moderate or severe renal impairment at baseline [112].

Weekly exenatide against placebo was examined in the EXCEL Trial (Exenatide), which studied T2D patients with a history of cardiovascular disease (CVD). The study reported a significant reduction in incident macroalbuminuria, although no other significant renal outcomes were affected. REWIND Trial with Dulaglutide treatment over 5.4 years was associated with a significant reduction in renal composite outcomes (new macroalbuminuria, a sustained decline in eGFR of 30% or more from baseline, or chronic renal replacement therapy), although the absolute differences in eGFR by the end of the study were minor [113,114].

AWARD-7 Trial compared the effects of dulaglutide with insulin glargine in T2D patients with moderate to severe CKD. Dulaglutide led to a lower decline in eGFR and reduced UACR, particularly in patients with macroalbuminuria at baseline [115]. Importantly, the PIONEER-5 Trial, apart from the safety and efficacy of oral semaglutide in glycemic control, showed that this drug did not alter eGFR but effectively lowered UACR in T2D patients with eGFR between 30–59 mL/min/1.73 m^2^ [116].

In a comprehensive meta-analysis incorporating new data from the AMPLITUDE-O trial, researchers examined the efficacy of GLP-1RAs in the treatment of T2D patients. This study utilized a random effects model to analyze data from eight large-scale trials involving 60,080 patients to assess the impact of GLP-1RAs on major adverse cardiovascular events (MACE), all-cause mortality, hospital admissions for heart failure, and various kidney outcomes. The analysis revealed that GLP-1RAs reduced the risk of MACE by 14%, all-cause mortality by 12%, hospital admissions for heart failure by 11%, and composite kidney outcomes by 21%. These kidney outcomes included the development of macroalbuminuria, a significant decline in eGFR, the requirement for kidney replacement therapy, or death due to kidney disease. Additionally, the treatment showed a positive effect on worsening kidney function based on eGFR changes. Notably, these benefits were consistent across various subgroups, including patients with or without cardiovascular disease, different levels of baseline HbA1c, and varying eGFR. The study highlighted the consistent efficacy of GLP-1RAs in reducing cardiovascular and renal risks in T2D patients, irrespective of the drug’s structural homology to human GLP-1 or exendin-4. The safety profile of GLP-1RAs was favorable, with no increase in the risk of severe hypoglycemia, retinopathy, pancreatitis, or pancreatic cancer compared to the placebo. Thus, this analysis underscores the broad applicability and potential of GLP-1RAs in managing cardiovascular and renal outcomes in T2D patients [117].

Recently, the FLOW trial, with a primary focus on the renal outcomes with semaglutide in T2DM patients with CKD, was prematurely stopped due to compelling evidence of renal protection. This decision, announced by Novo Nordisk on 10 October 2023, was based on the recommendation of the independent Data Monitoring Committee after a pre-specified temporary analysis met the criteria for early termination due to the shown efficacy. Designed as a randomized, double-blind parallel-group, event-driven, phase 3b superiority trial, FLOW compared subcutaneous semaglutide 1.0 mg with a visually identical placebo alongside standard care in patients with T2DM and CKD. The trial included a dose-escalation regimen starting from 0.25 mg/week, increasing to 0.5 mg, and then to a maintenance dose of 1.0 mg/week. In total, 3534 patients were enrolled across 28 countries. The primary objective was to demonstrate a delay in the progression of CKD and a reduction in the risk of renal and cardiovascular mortality. The composite primary endpoint included the onset of kidney failure (defined as chronic kidney replacement therapy or persistent eGFR <15 mL/min/1.73 m^2^), death from kidney failure, cardiovascular death, and the onset of a persistent ≥50% reduction in eGFR from baseline. Key secondary endpoints encompassed the annual rate of change in eGFR, MACEs, and all-cause death [118].

The early cessation of the FLOW trial highlights the potential of semaglutide as a pharmacotherapy option for patients with T2DM and CKD to prevent clinically relevant renal outcomes. The results of this study are eagerly anticipated by the medical community, given their potential to change clinical practice in managing patients with T2DM and CKD.

Recently, there has been real-world evidence on the effects of GLP-1 receptor agonists (GLP-1RAs) and long-acting insulins (LAIs) on kidney health in type 2 diabetes patients who need intensive blood sugar control and have a high risk for CKD progression. Analyzing data from 7279 matched pairs from Taiwan’s National Health Insurance Research Database found that GLP-1RAs significantly reduced the risk of severe kidney outcomes, including renal failure and the need for dialysis, compared to LAIs. The findings suggest that GLP-1RAs offer superior kidney protection for these patients and indicate a more significant benefit for those with cardiovascular disease or good adherence to oral diabetes medications [119]. Table 2 outlines the impact of GLP-1 receptor agonists (GLP-1RAs) on reducing albumin in the urine and protecting kidney function in type 2 diabetes (T2D).

Finally, the ongoing GLP-1 receptor agonist (GLP-1RAs) trials are predicting a new era in the treatment of chronic kidney disease (CKD) and cardiovascular disease (CVD) in type 2 diabetes (T2D). The REMODEL study aims to explore how semaglutide affects kidney health, thereby enhancing CKD treatments with innovative mechanistic insights. Additionally, the SOUL trial like Flow is evaluating the effectiveness of semaglutide in reducing severe renal outcomes and cardiovascular mortality in T2D patients, potentially establishing GLP1-RAs as foundational in protecting both kidney and heart health. The SELECT trial is venturing into using semaglutide for patients with atherosclerotic cardiovascular disease (ASCVD) and obesity, suggesting a broader role for GLP1-RAs beyond diabetes management, extending into cardiovascular prevention [120,121]. Moreover, the SURPASS-CVOT [121,122] and TREASURE-CKD trials are investigating the benefits of tirzepatide, which provides dual GIP and GLP1 receptor activation, offering enhanced benefits in glycemic control, weight reduction, and organ protection compared to GLP1-RA monotherapy. These trials are expected to significantly inform new clinical guidelines, providing a robust evidence base for incorporating GLP1-RAs into treatment regimens for T2D, CKD, and CVD. This comprehensive research effort is crucial for understanding and applying GLP1-RAs in managing chronic conditions, potentially leading to improved patient outcomes and quality of life.

In summary, while GLP1-RA has demonstrated beneficial effects on kidney outcomes in patients with T2D, further studies are needed to fully elucidate their impact on severe kidney outcomes such as eGFR decline, progression to ESKD, and kidney-related death.

## 4. Conclusions

The molecular pathways through which GLP1-RA confer renal protection in T2DM and DKD are complex and multifaceted. They include the modulation of renal hemodynamics, antioxidative and anti-inflammatory actions, metabolic regulation, and direct cellular effects. These mechanisms highlight GLP1-RA’s potential as a therapeutic option for glycemic control and direct and indirect renal function protection in diabetic patients. Ongoing research promises to enhance renal outcomes in T2DM and expand GLP1-RA’s therapeutic applications.

In conclusion, optimal glycemic control remains a critical strategy for slowing the progression of CKD in diabetes, with significant effects on reduction in hyperfiltration and albuminuria. Integrating GLP-1RAs into the therapeutic regimen offers a promising adjunctive approach, contributing to renal protective strategies against diabetes. The evolving evidence supports a multifaceted approach to managing DKD, highlighting the importance of both traditional and novel therapeutic modalities in preserving kidney health.

## Figures and Tables

**Table 1:** GLP-1 receptor agonists: bridging antioxidative and anti-inflammatory mechanisms in diabetes, atherosclerosis, and kidney function.

Effects	Pathway	Outcome
Antioxidative Stress Mechanisms	GLP-1RAs activate receptor-mediated pathways (cAMP, PI3K, PKC), leading to the activation of Nrf-2.	Enhanced activity of antioxidant enzymes (superoxide dismutase, glutathione reductase, catalase), reduction in oxidative stress markers, and protection against oxidative damage in pancreatic cells, liver, and cardiac tissues [5,15,76,77,78].
Anti-inflammatory Effects	GLP-1RAs modulate the immune system, reducing proinflammatory cytokine and chemokine levels (TNF-alpha, MCP-1/CCL2, IL-6, IL-1β), and decreasing macrophage infiltration in renal and vascular tissues.	Suppression of inflammation in diabetic nephropathy and atherosclerosis, protection against kidney and cardiovascular diseases [5,15,86,87,101,102].
Renal Protection	Restoration of critical enzymes for oxidative stress protection (catalase, glutathione peroxidase-3), inhibition of superoxide generation and NADPH oxidase activity.	Decreased risk of CKD progression, protection against diabetic nephropathy [79,80,81,82,83,84,85].
Insulin Sensitivity and Lipid Metabolism	GLP-1RAs improve lipid metabolism by reducing lipogenesis, inhibiting lipid peroxidation, and enhancing fatty acid β-oxidation. They also enhance insulin sensitivity by reducing the production of inflammatory cytokines in adipose tissue.	Reduced lipotoxicity and improved lipid homeostasis Improved lipid homeostasis, reduced insulin resistance, and therapeutic benefits extending beyond glycemic control [90,91,92,93,94,95].
Cardiovascular Protection	Reduction in endothelial dysfunction, attenuation of microvascular permeability, and reduced expression of adhesion molecules. GLP-1RAs inhibit oxidative stress in endothelial cells and reduce atherosclerotic lesions through AMPK and MAPK-dependent mechanisms.	Lowered progression of atherosclerosis, reduced cardiovascular risks, and improved endothelial function [53,87,88,89,96,97,98,99,100].
Direct Anti-Atherosclerotic Action	GLP-1RAs directly influence atherosclerotic plaque development and stability, reducing foam cell formation and carotid intima-media thickness, and affecting the formation and progression of early-stage atherosclerosis.	Stabilization of existing plaques, improved endothelial function, and reduced atherosclerosis progression [96,97,98,99,100].

The table illustrates the effects of GLP-1 receptor agonists on atherosclerosis through several pathways. These include anti-inflammatory and antioxidant mechanisms, kidney and heart protection, and enhancements in lipid metabolism and insulin sensitivity. These effects contribute to the protection of the heart and kidneys. References are provided to support these findings.

**Table 2:** GLP-1 receptor agonists (GLP-1RAs) effect on albuminuria and GFR protection in type 2 diabetes (T2D).

	GLP-1RA Used	Population Characteristics	Duration	Key Findings on Renal Outcomes
ELIXA	Lixisenatide	T2D patients with recent acute coronary events	108 weeks	Significant reduction in UACR; no significant change in eGFR or other hard renal endpoints [111]
LEADER	Liraglutide	Individuals with T2D and high cardiovascular risk	3.8 years	Significant reductions in macroalbuminuria; slightly slower decline in eGFR compared to placebo, more pronounced in patients with moderate or severe renal impairment [112]
SUSTAIN-6	Semaglutide	Individuals with T2D and high cardiovascular risk	104 weeks	58.6% of the UACR lowering effect independent of body weight and HbA1c; significant reductions in macroalbuminuria; slightly slower decline in eGFR compared to placebo [112]
EXSCEL	Exenatide	T2D patients with a history of cardiovascular disease (CVD)	Mean 3.2 years	Reduced progression to microalbuminuria (*p* = 0.02); for the 40% eGFR decline + ESRD endpoint, the predicted and observed risk reductions were 11.0% (HR 0.89; 0.82–0.97) and 13.7% (HR 0.86, 0.72–1.04), respectively [113]
REWIND	Dulaglutide	T2D patients, longer-term study	5.4 years	Reduced albuminuria and surrogate renal endpoints; a ≥40% sustained eGFR decline occurred less frequently among participants assigned to DU than placebo (HR 0.72, 95% CI 0.58–0.88, *p* = 0.002); the mean annual decline in eGFR slope was significantly smaller for participants assigned to DU than placebo (−1.37 vs. −1.56 mL/min/1.73 m^2^/year, *p* < 0.001) [113,114]
AWARD-7	Dulaglutide	T2D patients with moderate to severe CKD	52 weeks	Significantly lower decline in eGFR; reduced UACR, particularly in patients with macroalbuminuria at baseline [115]
PIONEER-5	Oral Semaglutide	T2D patients with eGFR between 30–59 mL/min/1.73 m^2^	52 weeks	No alteration in eGFR; effective reduction in UACR [116]
Meta-analysis including AMPLITUDE-O trial	Various GLP-1RAs	Large-scale trials involving 60,080 patients with T2D	Varied across studies	GLP-1RAs reduced the risk composite kidney outcomes by 21% [117].
FLOW	Semaglutide	T2DM patients with CKD	(Trial halted early due to efficacy)	Early termination due to compelling evidence of renal protection; awaiting detailed results [118].

Summary of GLP-1 receptor agonist (GLP-1RA) trials on renal outcomes in T2DM patients. References are provided to support these findings.
